# From Motion to Emotion: Accelerometer Data Predict Subjective Experience of Music

**DOI:** 10.1371/journal.pone.0154360

**Published:** 2016-07-14

**Authors:** Melanie Irrgang, Hauke Egermann

**Affiliations:** 1 Audio Communication Group, Technische Universität Berlin, Berlin, Germany; 2 Department of Music, University of York, York, United Kingdom; Champalimaud Foundation, PORTUGAL

## Abstract

Music is often discussed to be emotional because it reflects expressive movements in audible form. Thus, a valid approach to measure musical emotion could be to assess movement stimulated by music. In two experiments we evaluated the discriminative power of mobile-device generated acceleration data produced by free movement during music listening for the prediction of ratings on the Geneva Emotion Music Scales (GEMS-9). The quality of prediction for different dimensions of GEMS varied between experiments for *tenderness* (R_1_^2^(first experiment) = 0.50, R_2_^2^(second experiment) = 0.39), *nostalgia* (R_1_^2^ = 0.42, R_2_^2^ = 0.30), *wonder* (R_1_^2^ = 0.25, R_2_^2^ = 0.34), *sadness* (R_1_^2^ = 0.24, R_2_^2^ = 0.35), *peacefulness* (R_1_^2^ = 0.20, R_2_^2^ = 0.35) and *joy* (R_1_^2^ = 0.19, R_2_^2^ = 0.33) and *transcendence* (R_1_^2^ = 0.14, R_2_^2^ = 0.00). For others like *power* (R_1_^2^ = 0.42, R_2_^2^ = 0.49) and *tension* (R_1_^2^ = 0.28, R_2_^2^ = 0.27) results could be almost reproduced. Furthermore, we extracted two principle components from GEMS ratings, one representing arousal and the other one valence of the experienced feeling. Both qualities, arousal and valence, could be predicted by acceleration data, indicating, that they provide information on the quantity and quality of experience. On the one hand, these findings show how music-evoked movement patterns relate to music-evoked feelings. On the other hand, they contribute to integrate findings from the field of embodied music cognition into music recommender systems.

## Introduction

Music is often used to regulate emotions like reducing stress or to influence one’s mood as shown by [1] or [2]. This means that listening to music is highly linked to the experience of emotions [3–5]. Nonetheless, these subjective qualities of music do still play a minor role in the field of Music Information Retrieval (MIR) and Music Recommender Systems (MRS), i.e. for the retrieval and recommendation of music offered by web-based services [6].

In the field of *Affective Computing* [6] there is increasing effort to connect the physical characteristics of music to emotional *valence* and *arousal* values of the circumplex model from [7]. However, considering acoustical features of music and its implications on the perception of emotions does not yet consider the *motor origin hypothesis* of emotion in music as claimed by [8]. Accordingly, music is often discussed to be emotional because it reflects expressive movements in audible form [9–11]. Furthermore, the term *emotio* derived from the Latin *movere*, to move, and is being used as a synonym for *being moved*. That is why Leman calls for new non-verbal, embodied possibilities to describe music and its experience [12]. He suggests to use corporeal articulations as a bridge between linguistic self-report measures and measurements of physical energy like *pitch*, *loudness* or *tempo* because “human action can realize the transformation from physical energy to cultural abstraction, and vice versa” ([12] p. 77).

Describing music by moving a mobile device like a smartphone, could build a bridge between physical energy and subjective experience. Smartphones are among the most increasingly popular devices to listen to music [13]. Once corporeal articulations are available from smartphone-assessed motion data and emotionally interpretable, missing emotional descriptions of music in MIR and MRS could be provided based on these embodied descriptions. Hence, a model able to translate between corporeal and verbal descriptions of music would not only offer innovative, multimodal access to the retrieval of music, but also place additional semantic annotations about the emotional qualities of music at the disposal that also enrich the conventional verbal search.

## 1 Verbal Models of Musical Emotion

The most widespread models to describe the emotional qualities of music listening experience are the *basic emotions model*, the *circumplex model* and the *Geneva Emotion Music Scales (GEMS)* [14].

The basic emotion model assumes, that there are four to six basic emotions that have evolutionary meaning and that are culturally universal. They often include *fear*, *anger*, *happiness*, *sadness*, *disgust*, and *surprise* [14]. The circumplex model maps these basic emotions and many other emotional feelings onto a two-dimensional space that is spanned by *valence* (“how pleasant or unpleasant is the experience?”) and *arousal* (“how intense is the experience?”) [7]. Accordingly, the quality of each emotion can be described by these two underlying qualities.

The GEMS have been iteratively developed and evaluated in four studies [15]. The aim for music-related emotions was to find “a more nuanced affect vocabulary and taxonomy than is provided by current scales and models of emotion” ([15] p. 513). The original version of the GEMS comprises 45 terms including *feeling of transcendence*, *nostalgic*, *solemn* or *impatient* that are not part of any other emotional model. The GEMS-9, a shortened version of the GEMS-45, are, like the long-version, grouped into the categories of *sublimity* (wonder, transcendence, tenderness, nostalgia, peacefulness), *vitality* (power, joyful activation) and *unease* (tension, sadness) [15]. Torres-Eliard, Labbé and Grandjean collected self-report measures of the GEMS and suggest that it was a suitable model to assess musical emotion [16]. They concluded that “the results indicate a high reliability between listeners for different musical excerpts and for different contexts of listening including concerts, i.e. a social context, and laboratory experiments” ([16] p. 252).

## 2 Corporeal Articulation of Musical Emotion

Several studies have shown that there is a close link between movement and emotion in music. Sievers et al. asked participants to adjust the features *rate*, *jitter*, *consonance/smoothness*, *step size* and *direction* for each of the following five emotions: *anger*, *happiness*, *peacefulness*, *sadness* and *fear* [10]. For one group the adjustment of features led to different movement and appearance of a bouncing ball. For the second group adjusting features changed the melody and expression of a piano piece. Experiments were conducted both in the U.S.A. and Cambodia. The settings used for different emotions were highly similar for motion and music in both cultures. Accordingly, the authors conclude that emotion expression in music and movement seem to be based on the same universal features. Giordano et al. studied the relationship between walking and emotion and its implications for the expression of musical performances [8]. Slow, quiet, and irregular walking sounds were associated with expressing sadness while fast, loud, and regular walking sounds with happiness. Similar patterns are also used in music performance (see [17]). Thus, they concluded that their findings “support the motor-origin hypothesis of musical emotion expression that states that musicians and listeners make use of general movement knowledge when expressing and recognizing emotions in music” ([8] p. 29). This connection between musical rhythm and motor activities was supported by Parncutt already in 1987 [18].

There are many possibilities to express music listening experience in an embodied way. Among them are tapping or moving parts of the body along with the beat, singing, imitating to play a musical instrument or dancing. Hedder also evaluated an approach based on facial expression as form of embodiment [19]. Drawing as described in De Bruyn, Moelants, and Leman [20] and [12] is another alternative as a means of graphical attuning to the experience. Last but not least, the employment of acceleration sensor data generated by arm gestures as by Amelynck, Grachten, van Noorden and Leman [21] was described to be a very promising approach of multimodal querying on mobile devices.

Amelynck et al. [21] investigated how motion can be linked to emotion in the context of MIR. They asked participants to perform arm gestures while holding a Wii remote controller in order to describe the music. Afterwards the emotional qualities of the musical excerpts were rated on the dimensions of *valence* and *arousal*. Using motion features recorded with the Wii controller generated fairly good predictions for the dimension of *arousal*, but performed less precisely for the dimension of *valence*. The authors argue that this might be due to people rating *sad* music as pleasant as described in [22]), and conclude that the circumplex model might be unsuitable to be used with musical emotions. Also Juslin and Vjästfjäll note that perceiving mixed emotions, that are positive and negative at the same time, limits that employability of the circumplex model [23]. Accordingly, Amelynck et al. suggest the GEMS as an alternative to this circumplex model.

### Aims and Experimental Design

The goal of the presented study is to explore which and how well each of the GEMS can be predicted by mobile-device generated acceleration data. That way, the present study continues the work of Amelynck [21], testing the use of an alternative emotion model and using different motion sensors. Hence, these findings will contribute to understand, how acceleration data can be used to integrate embodied music cognition into Music Recommender Systems. Furthermore, it will test the often described similarities between certain motion features and emotional qualities [8][10].

First, a pilot study was conducted to develop a measurement instrument for the following experiments. Afterwards, we conducted two experiments to test if and how accelerometer data can be used to describe musical experience. Here, the second experiment tested if results could be replicated for different music samples and if results changed when participants were free to choose the songs they felt like moving to. Furthermore, we also tested how different GEMS qualities relate to different movement patterns (rhythmic vs. gestalt), e.g. if music experienced as *sad* was less suitable for rhythmic movement patterns.

## General Methods

### Ethics Statement

Prior to participating in both experiments, individuals were informed of their general goals and of the procedures involved, i.e. describing the music corporeally and to rate its emotional qualities. They gave oral consent to participating in the study and storing of the data collected during the experiment. No ethics approval was required from the Technical University Berlin for behavioral studies such as those reported in this manuscript. There was no institutional review board available at the department where the experiment was conducted. Neither of the experiments involved deception or stressful procedures. Participants were informed that they were free to leave the experiment at any time, and that their data was analyzed anonymously. Participants in both experiments were recruited on a voluntary basis from the students and acquainted interested persons. Some students got course credit for participation. Others shared a professional or private interest in the study and its methodology and therefore volunteered to participate. The research reported in this manuscript was carried out according to the principles expressed in the Declaration of Helsinki. No other than the personal identifying information reported in this manuscript were collected (see Methods section).

### Development of Measurement Smartphone App

We conducted a pilot study to design and accompany the development of the measurement instrument. In this phase, we interviewed 11 persons with different backgrounds, i.e. different age, gender, musicians and non-musicians, for their preferred way of describing music experience in an embodied way. After testing two favorites in a prototypical stage, participants opted for performing free movements while holding their smartphone device over drawing lines. Hence, an Android App was developed iteratively applying the think-aloud method to integrate participants’ feedback as described by [24].

The choice for Android was due to a wider spread of Android devices that could enable us to repeat the experiment with a lager sample size in the future. The app presented the music stimuli to participants and simultaneously recorded accelerometer data from smartphone sensors (for more details, we want to refer to the documentation for Android Developers [25]). Afterwards, it presented nine emotional attributes taken from the GEMS-9 short version. Here, participants were instructed to rate the emotional qualities of the music excerpts presented. Screenshots showing the App are available as Supporting Information S2 Fig.

### Stimulus Selection

Music used in both experiments was selected in a participatory approach [26] based on suggestions by the participants of the pilot study according to the following criteria:

1. account for a variety of field participants’ preferences
2. cover the range of the GEMS-9
3. keep the balance between *female* and *male* artists
4. do not let emotions be covered in a stereotypical way like *tenderness* by *female* artists or *tension* by *male* artists
5. artists of different color
6. cover a variety of genres

For each musical piece, an excerpt of ~40s duration was chosen such that it was as homogeneous as possible w.r.t GEMS qualities during this time.

### Motion Data Analysis

The general workflow for motion data analysis was as follows:

1. get raw acceleration data from motion sensor for x, y and z in 3D space
2. cut beginning (first 5s) and end to standardize duration d of signals to d = 35s
3. resample with sample rate ~5.7Hz
4. apply PCA to x, y and z
5. extract motion features
6. normalize range of motion features intra-individually
7. split data set into training (50%) and test (50%) set
8. stepwise select features on training set and fit linear regression model for each GEMS feeling
9. evaluate model quality on test set for each GEMS feeling

#### Motion Feature Extraction

**Fig 1:**
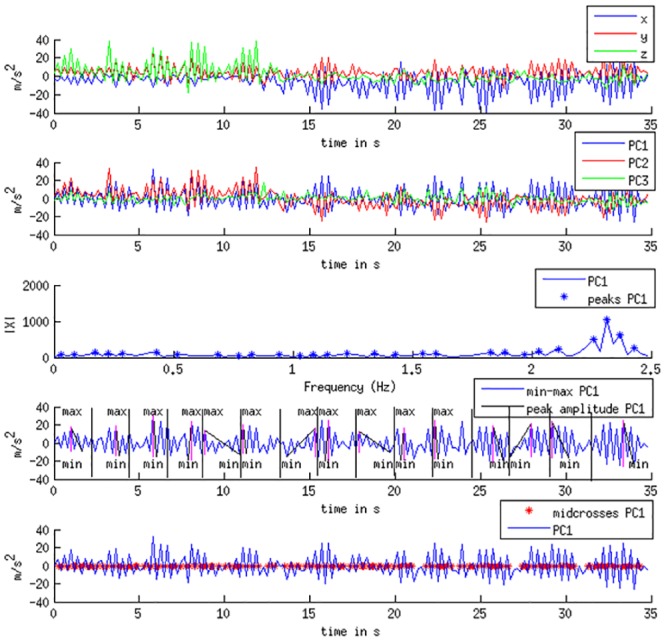
Example of Extracted Features for the Movements of one Participant to the Music Excerpt *Wargasm*, Experiment 1: The figure shows a participant performing large and fast movements. After about 12s the direction of movement changed, probably for ergonomic reasons (first subplot). Principle components of accelerometer data are shown in the second subplot. The third subplot indicates that the maximum frequency magnitude lies at a high frequency. The fourth subplot visualizes the computation of the signals’ amplitudes (only for PC1). The stars in the fifth subplot show the midcrosses of the motion signal.

As participants needed a few seconds to fully get into the movement, the first five seconds were cut from the beginning of the motion data. Prior to feature extraction we applied a *Principle Component Analysis* (PCA) to each recording of accelerometer data (per stimulus and participant). That way, the x-, y- and z-dimensions were transformed to three principal components PC1, PC2 and PC3. This helped to enhance the comparability of movements between participants, e.g. to account for different ways of holding a device. We did not apply PCA in order to compress data, all dimensions were kept. Furthermore, we did not extract direction-relevant features that would have spoken against applying a PCA. Table 1 shows an overview on the features extracted to characterize the movement, categorized into *tempo*, *size*, *regularity* and *smoothness*. The statistical features *absolute skewness*, *median* and *standard deviation (std)* are computed to get a time compressed representation of the features extracted from the time series of motion data. As most features were not normally distributed, we chose to compute the *median* over the *mean*. During the selection of features we learned that the *standard deviation* still served as a significant feature to represent the degree of variance in the distribution. In order to remove any inter-individual differences in movement size, all features were subsequently range-normalized intra-individually to the interval [0-1]. Fig 1 shows an example of one accelerometer recording before and after applying PCA. Here, the extraction of different features is illustrated. When results are described, we will refer to positive acceleration in eigenspace as *forward* and to negative acceleration in eigenspace as *backward* movement.

**Table 1:** List of Extracted Motion Features.

Feature	Description
**tempo**	
max_freq_hz	most dominant frequency in Hz across all three PCs
median_dist_midcrosses_PC1/2/3	median of distances between midcrosses
**size**	
median_peak_PC1/2/3	median amplitude
volume	volume of acceleration point cloud computed by applying delaunay triangulation to the acceleration data points in 3D-eigenspace and hence computing the convex hull of the triangulated space as in [21]
max_freq_mag	magnitude of most dominant frequency
**smoothness**	
median_rise_PC1/2/3	median duration of attacks
median_fall_PC1/2/3	median duration of releases
**regularity**	
max_freq_rel	magnitude of most dominant frequency relative to median magnitude of all frequency peaks
skewness_peak_PC1/2/3	absolute skewness of amplitude distribution
skewness_rise_PC1/2/3	absolute skewness of attacks’ (duration) distribution
skewness_fall_PC1/2/3	absolute skewness of releases’ (duration) distribution
skewness_dist_midcrosses_PC1/2/3	absolute skewness of midcrossings’ distribution
std_peak_PC1/2/3	std of amplitude distribution
std_dist_midcrosses_PC1/2/3	std of midcrossing’s distribution
std_rise_PC1/2/3	std of attacks’ (duration) distribution
std_fall_PC1/2/3	std of releases’ (duration) distribution
rel_max_freq_mag	magnitude of most dominant frequency relative to median of spectrum’s peak magnitudes

Notes: PC1/2/3 = first, second, and third principal component of movement data. For the computation of *amplitude* the data series is split into N = 15 segments. For each segment, Matlab’s peak2peak method is called to compute the absolute distance between minimum and maximum (see pink vertical line in fourth subplot of Fig 1). Midcrosses are the points in time when the signal crosses the mid reference level. They are computed by calling Matlab’s midcrosses function with a tolerance level of 45%. The rise and fall times, computed with Matlab’s risetime and falltime functions, indicate the time passed between one local minimum and the following local maximum (rise) and vice versa for fall. Volume of the acceleration data point cloud is computed by Matlab’s delaunayTriangulation and convexHull functions. Matlab’s Fast Fourier Function (fft) was applied to determine the most dominant frequency.

#### Model Fitting

We fitted one linear regression model for every GEMS feeling, with all motion features as predictor variables. There was no strong multi-collinearity between motion features indicated by the fact, that for any predictor, the *Variance Inflation Factor* was smaller than 10. Stimulus order effects could have resulted in an *emotional afterglow* effect of stimuli (e.g. the rating of the second piece is influenced by the rating of the first one), violating the assumption of observational independence in linear regression models. However, no such effect could be observed in test plots on autocorrelation of residuals and test plots mapping *order* against residuals. Features were selected using the forward stepwise algorithm for linear regression [27]. Before selecting the features and fitting the model, the data was partitioned into training and test set, ~50% each, in order to evaluate each model’s ability to generalize with unseen data. The test set was compiled by randomly selecting five observations from each participant.

## Experiment 1

### 2.1 Method

The following sections summarize the first experiment that was conducted after developing the measurement app in the pilot study.

#### Stimuli

The musical stimuli for the first experiment were compiled by the pilot study’s participants according to the criteria described in the General Methods section. Table 2 shows the final list of musical excerpts. The list of samples was presented in random order for each participant. However, they were then free to choose the preferred order in which to assess the samples.

**Table 2:** List of Music Excerpts First Experiment.

Artist/Band	Title	Genre
L7	Wargasm	Punk
Souad Massi	Raoui	Folk Rock
Adele	Rolling in the Deep	Pop
Bruno Mars	Count on Me	Pop
Air	La Femme d’Argent	Electronica
Two Fingers	Sweden	Grime
Bob Marley	Corner Stone	Reggae
Mel Bonis	Berceuse Op.23 No.1	Classic
Andy Allo	People Pleaser	Funk
David Bowie	Rebel Rebel	Rock’n Roll

#### Participants

For this experiment, we recruited 22 participants from the Master of Science program *Audio Communication and Technology* at TU Berlin. They had an average age of 27 years (SD = 2.36). 73% identified as ‘male’, 18% as ‘female’, 5% as ‘rather male’ and another 5% did not identify with any gender. 91% were experienced in playing an instrument, the production of music or singing in a choir. 5% only had short term experience in making music beyond classes in school and 5% indicated to have no experience at all. 36% already participated in dancing classes or similar activities for which movement is related to music. 50% were only dancing occasionally in clubs or on concerts. 14% had no experience at all in moving to music. 82% are regularly using a smartphone, 9% are experienced in using a smartphone but do not use one now and 9% are not using one at all. Participants also indicated, that for them they most formative music genres were Rock* (68%), Electro* (55%), Hip Hop* (32%), Classic (27%), Metal (22%), Jazz (23%), Punk (23%), Pop* (18%), Dub(step) (18%) and Reggae (14%). (Note: *several similar subgenres were grouped to one).

#### Procedure

The laboratory used was illuminated only slightly and offered enough space to move freely. The app ran on a Motorola Moto G with Android Version 4.4. Participants wore AKG headphones featuring a 1,5m long cable. During the experiment, they were alone in the lab with doors closed. Before, a guided test tour through the app was given in order to familiarize participants with the experiment. They were informed that the study was about describing the music corporeally and to rate it in terms of the GEMS. They did not know that, subsequently their GEMS ratings would be predicted from movement. We also told them that there was no right or wrong way to move to the music.

After a participant selected a song, the first step was to listen to the song in order to be prepared for the corporeal articulation. Participants could stop the presentation of the excerpt early when they decided that they knew the music already well enough to describe it.

Afterwards, the movements were actually recorded by the device’s acceleration sensor synchronized to the music. For this part of the study participants were instructed as follows: “Please move now with the device according to the music. It is important that you stand and don’t sit during motion capturing. You can move freely, i.e. all parts of the body, but keep in mind that only movement of the device can be captured.”

After each embodied description participants rated the perceived emotional qualities of the musical excerpts according to the GEMS-9 on a 100-point, unipolar intensity scale initialized to ‘0’ (Table 3). They were instructed as follows: “Please rate the perceived emotional quality of the music according to the GEMS-9. Do not rate how you *felt* during listening.” Subsequent to the GEMS, participants were asked how suitable they considered both embodied and verbal descriptions for the music excerpt. At the end of the experiment participants were asked to fill out a short socio-biographical questionnaire.

**Table 3:** Emotion Ratings Items: Participants rated their experience of nine German GEMS categories (each defined by three additional adjectives).

Original English	German translation
**wonder**	**Verwunderung**
filled with wonder, dazzled,	voller Verwunderung, geblendet,
allured, moved	verlockend, bewegt
**transcendence**	**Transzendenz**
fascinated, overwhelmed,	fasziniert, überwältigt,
feelings of transcendence and spirituality	Gefühl von Transzendenz und Spiritualität
**power**	**Energie**
strong, triumphant, energetic, fiery	stark, triumphierend, energetisch, glühend
**tenderness**	**Zärtlichkeit**
tender, affectionate, in love, mellowed	liebevoll, zärtlich, verliebt, weich
**nostalgia**	**Nostalgie**
nostalgic, dreamy, sentimental,	nostalgisch, verträumt, sentimental, melancholisch
**peacefulness**	**Friedlichkeit**
serene, calm, soothed, relaxed	still, ruhig, sanft, entspannt
**joyful activation**	**Freude**
joyful, amused, animated, bouncy	freudig, vergnügt, lebhaft, hüpfend
**sadness**	**Traurigkeit**
sad, sorrowful	traurig, sorgenvoll
**tension**	**Anspannung**
tense, agitated, nervous, irritated	angespannt, aufgeregt, nervös, irritiert

### Results and Discussion

#### Similarities between Movements and GEMS for First Experiment

The fixed effects from Table 4 indicate that music perceived as *transcendent* was related to a rather irregular tempo of movement (std_dist_midcrosses). For *wonder* the movement’s size (std_peak) was irregular in the second component and regular (skewness_peak) in the first principle component. *Power* related to regular and large movements. *Tenderness* was described by small (median_peak) movements with regular backward (std_fall) and irregular forward (std_rise) gestures. *Nostalgia* was also described by small movements (median_peak) with irregularly smooth backward movements in the third component and regular ones in the second component (skewness_fall). In contrast to *power*, *peacefulness* is characterized by small movements. When participants rated music as *joyful*, they performed movements with regular backward phases and less regular forward movements like jumping. For *sadness* movements were slow while for *tension* movements were primarily large with irregular tempo.

**Table 4:** Fixed Effects Modeling Parameter Estimates for GEMS Ratings and their Principle Components of First Experiment Predicted via Motion Features Related to *Size*, *Rate*, *Regularity* and *Smoothness* of the Movement.

fixed effects	estimate	SE	t-statistic	p-value
GEMS-9				
**transcendence**				
intercept	18.1	4.5	4.0	.0002
std_dist_midcrosses_PC1	32.5	11.1	2.9	.0050
**wonder**				
intercept	20.7	6.0	3.4	.0012
skewness_peak_PC1	-22.9	9.8	-2.3	.0228
std_peak_PC2	36.7	9.6	3.8	.0004
**power**				
intercept	21.1	5.6	3.7	.0005
median_peak_PC1	70.3	11.6	6.1	<.0001
std_peak_PC3	-29.5	12.1	-2.4	.0185
**tenderness**				
intercept	69.9	6.1	11.5	<.0001
median_peak_PC1	-67.5	9.8	-6.9	<.0001
std_rise_PC3	38.4	12.1	3.2	.0025
std_fall_PC3	-32.6	14.0	-2.3	.0241
**nostalgia**				
intercept	59.9	7.9	7.6	<.0001
median_peak_PC1	-57.1	10.6	-5.4	<.0001
skewness_fall_PC2	-23.0	10.5	-2.2	.0324
skewness_fall_PC3	28.4	11.2	2.5	.0147
**peacefulness**				
intercept	60.3	7.3	8.3	<.0001
median_peak_PC1	-46.5	13.0	-3.6	.0007
**joyful activation**				
intercept	42.7	4.5	9.5	<.0001
std_rise_PC3	42.8	12.4	3.5	.0011
std_fall_PC3	-39.6	14.6	-2.7	.0089
**sadness**				
intercept	11.6	4.0	2.9	.0057
median_dist__midcrosses_PC1	36.3	8.9	4.1	.0002
**tension**				
intercept	-8.9	7.9	-1.1	.2649
std_dist_midcrosses_PC1	28.7	10.9	2.6	.0114
median_peak_PC1	30.5	10.1	3.0	.0040
skewness_fall_PC3	23.5	10.2	2.3	.0255
Principle Components of GEMS-9				
**PC-1 relaxation**				
intercept	1.0	.2	5.6	<.0001
median_peak_PC1	-2.1	.3	-6.4	<.0001
**PC-2 positive valence**				
intercept	0.3	.2	1.8	.0733
median_dist_midcrosses_PC2	-0.8	.4	-2.2	.0035

**Fig 2:**
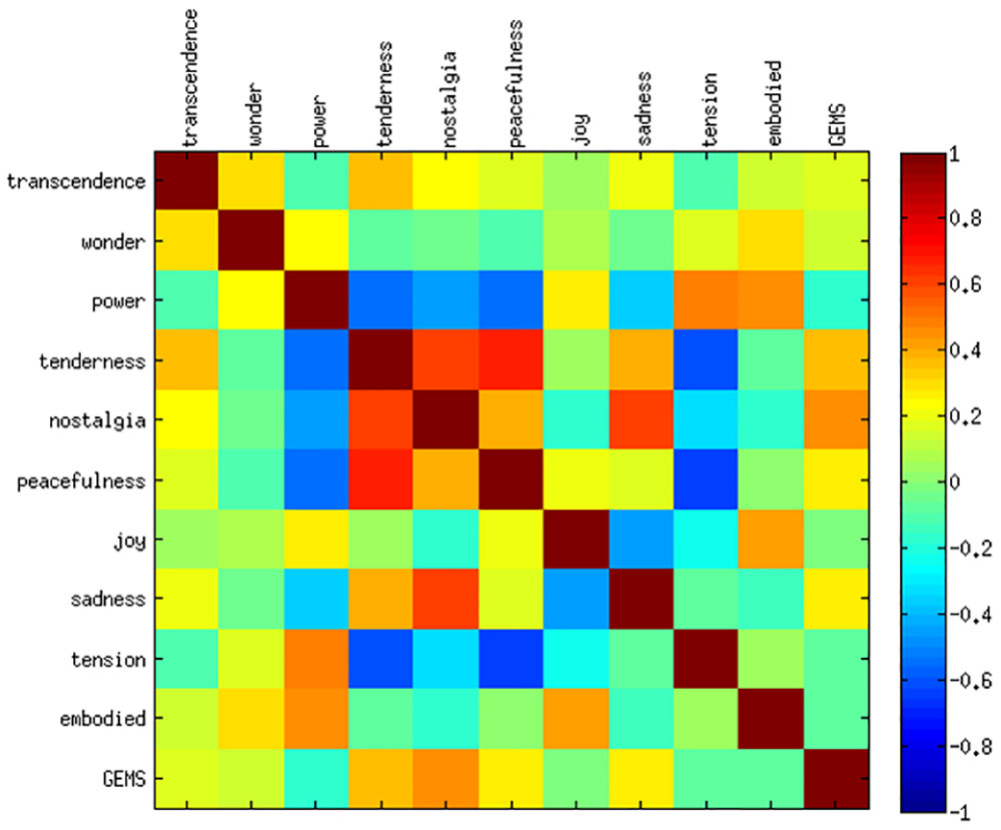
Spearman Correlation of GEMS and Description Preferences (Embodied vs. GEMS) for First Experiment.

Fig 2 shows that participants preferred rating on GEMS to describe their experience when they perceived *nostalgia, sadness, tenderness or peacefulness*. For *joy*, *power* and *wonder* participants preferred embodied descriptions. For *tension* and *transcendence* we did not observe such an association between feeling rating and description preference.

#### Prediction Results

Table 5 indicates that *tenderness*, *power* and *nostalgia* were predicted best, followed by *tension*, *wonder* and *sadness*. For *peacefulness* and *joy* around 20% of variance in the data could be explained by the fitted regression models. *Transcendence* was most difficult to predict because only 14% of the variance in the data could be explained through the motion data. Since for both, the training and test data set, a comparable amount of variance could be explained, there was no evidence for overfitting of the model to the data.

**Table 5:** R-Squared and RMSE for first experiment ranked according to their *R*^2^ on the training set.

	rmse (training)	*R*^2^ (training)	p-value (training)	rmse (test)	*R*^2^ (test)
**GEMS-9**					
tenderness	22.8	.497	<.0001	31.9	.732
power	22.8	.423	<.0001	26.2	.538
nostalgia	24.7	.423	<.0001	27.2	.582
tension	22.6	.284	.0006	27.9	.206
wonder	23.5	.247	.0006	24.4	.574
sadness	22.1	.238	.0002	21.2	.240
peacefulness	30.8	.196	.0007	32.9	.230
joyful activation	23.9	.193	.0038	32.8	.205
transcendence	24.0	.139	.0051	24.4	.275
**PCs of GEMS-9**					
PC-1 relaxation	0.8	.439	<.0001	0.8	.504
PC-2 positive valence	1.0	.082	.035	1.1	.079

Since we asked participants to rate the intensity of experience of the different GEMS, most feelings are likely to be correlated with the overall intensity or arousal of emotional experience (see Fig 2). Here, most feelings show small to large correlations with *power*. Therefore, we applied a PCA on these rating data, in order to represent emotional experience with less dimensions. A resulting arousal component in the GEMS rating could be interpreted as describing the quantity or intensity of the feeling, whereas a valence component as describing the quality. That way, we could identify, if there are potential differences in predicting the quantity/intensity and quality of emotional experience from movement data.

Based on the Elbow Method we extracted two components explaining most of the variance in the data. Table 6 visualizes the item loadings of the eigenvectors having the highest eigenvalues. The first PC might be interpreted as the degree of *relaxation* as it got high positive loadings for *tenderness*, *peacefulness* and *nostalgia*, but high negative loadings for *power* and *tension*. Accordingly, it represents the opposite of arousal. The second PC might be interpreted as *positive valence* as it got high positive loads for *joy* and *wonder*, but a negative loading for *sadness*. Hence, the interesting question was whether the motion features only predicted the degree of *relaxation* (vs. *arousal* and *activation*) or if they could also explain degrees of *valence* inherent in the experienced emotionality. The estimates for the fixed effects in Table 4 show that *relaxation* was expressed by small movements while *positive valence* related to fast movements. Furthermore, the prediction results from Table 5 indicate that for *relaxation* about 44% of the variance in data could be described by the fitted regression models, while only 8% *positive valence* variance is explained. As prediciton accuracy is similar on both training and test set, there was no overfitting of the model to the data.

**Table 6:** Principle Component Loadings of GEMS Ratings of First Experiment.

GEMS feeling	PC-1 Relaxation	PC-2 Positive Valence
tenderness	.889	.053
peacefulness	.841	.047
nostalgia	.754	-.203
power	-.690	.452
tension	-.687	-.060
transcendence	.474	.474
sadness	.470	-.373
joy	-.028	.790
wonder	-.070	.677

Notes: Components were rotated using the *varimax* method.

#### Discussion

**Fig 3:**
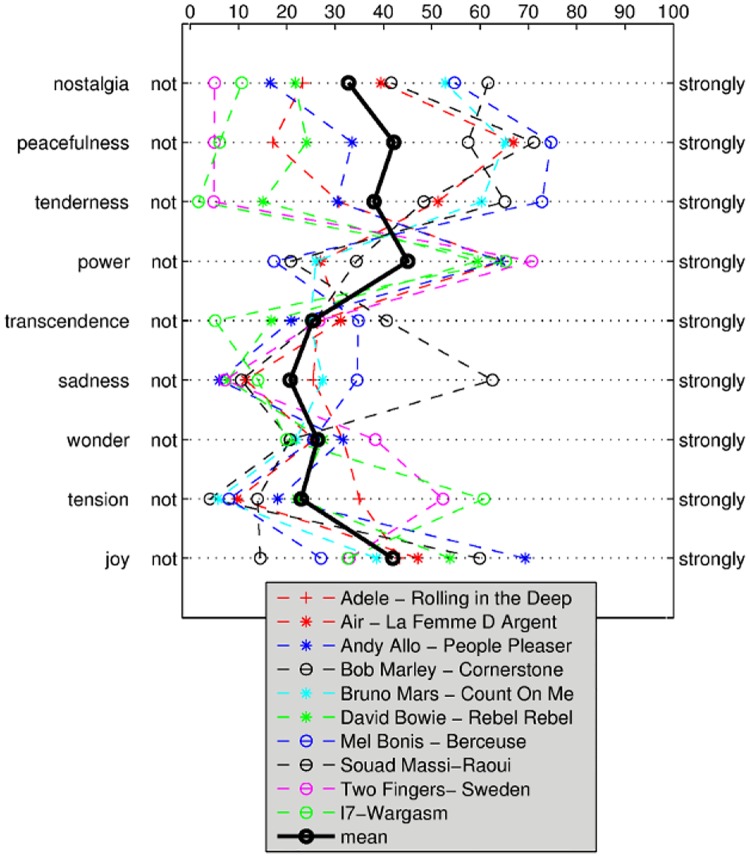
Polarity Profile of Mean GEMS Ratings for the First Experiement.

The findings in predicting the relaxation and valence components indicate, that not only arousal or intensity was predicted in the GEMS ratings, but also the quality of perceived emotion, i.e. *positive valence*. However, only 8% of the variance in valence were covered by the approach. Therefore, these results are similar to those of [21]. This could be explained by the fact there were several GEMS with little mean intensity and little variance as can be seen from the polarity profile in Fig 3. Examples are *transcendence*, *sadness*, *wonder*, and *tension*. Even though *joy* showed a rather high degree of variance according to the polarity profile, its prediction turned out to be difficult in this experiment. There might not have been a common movement pattern throughout participants for music perceived as *joyful*. In general, it also should to be noted that there have not been enough music excerpts to sufficiently cover all states of the GEMS. We therefore conducted a second experiment with a different set of stimuli that were chosen to cover more different GEMS feelings.

### Experiment 2

Experiment 2 was conducted in order to test, if the findings from Experiment 1 could be replicated. Furthermore, different music stimuli were chosen in order to cover more states of the GEMS. We also asked for more feedback concerning the movement patterns used from participants. Doing so, we tested the following two hypotheses: “Participants prefer embodied rhythmic-related descriptions when they perceive *power*, *tension* or *joy*” and “Participants prefer embodied gestalt-related descriptions or verbal descriptions (GEMS) when they perceive sublimity-related feelings like *nostalgia* or *transcendence*”. We assumed that sublimity-related musical emotions were more related to musical contour and melody. Thus, we assumed participants to imitate musical contour by performing gestures that are more gestalt-like and less rhythmic.

#### Stimuli

After the first experiment, during which participants were not free to choose the music they described corporeally, we thought it might further improve prediction results, if participants chose the music themselves. The study from Liljeström, Juslin and Västfjäll observed that emotions were perceived more intensely when participants chose the music themselves [28]. Therefore, we asked them to propose music in order to compile a list from which they could chose 10 samples out of 20. Table 7 shows the final list of musical excerpts used and how often participants chose each excerpt in absolute numbers.

**Table 7:** List of Music Excerpts for Second Experiment: Songs are ranked according to the frequency they were chosen by the participants.

Artist	Title	Genre	Frequency
Moby	Porcelain	Pop	17
Air	La Femme d’Argent	Electronica	16
Slipknot	Duality	Heavy Metal	15
Die Antwoord	I Fink U Freeky	Rap-Rave	14
M.I.A.	Paper Planes	Hip Hop	14
System of a Down	Roulette	Rock	14
Antony And The Johnsons	River Of Sorrow	Pop	14
Bartok	Allegro—Music for String Instruments Percussion and Celesta	Classic	13
The Beatles	In My Life	Pop	13
Chic	I Want Your Love	Disco	13
Teddybears STHLM	Little Stereo	Trip Hop	11
Miles Davis	Someday My Prince Will Come	Jazz	11
David Bowie	Rebel Rebel	Rock’n Roll	11
Adele	Rolling in the Deep	Pop	10
Caetano Veloso	Quem Me Dera	Alternative Rock	9
Souad Massi	Raoui	Folk Rock	8
Andy Allo	People Pleaser	Funk	8
LunchMoney Lewis	Bills	R&B	6
Mel Bonis	Berceuse Op.23 No.1	Classic	6
Helium Vola	Selig	Electro Medieval	5

#### Participants

21 students participated from the following courses: *Audio Communication and Technology* (40%), engineering in the field of technical environmental protection (20%), and others 40%. Participants had an average age of 28 years (SD = 3.0). 76% identified as ‘male’, 19% as ‘female’ and 5% did not identify with any gender. 72% were experienced in playing an instrument, the production of music or singing in a choir. 29% only had short term or no experience in making music beyond classes in school. 52% already participated in dancing classes or similar activities for which movement is related to music. 81% were only dancing occasionally in clubs or on concerts. 9% had only little or no experience at all in movement to music. 81% are regularly using a smartphone, 19% do not.

#### Procedure and Data Analysis

Procedure and data analysis were conducted as in the first experiment with the exception of the following minor changes:

1. All scales were initialized to ‘50’ instead of ‘0’ to equalize the effort to move the slider in either direction.
2. The German translation of the GEMS-9 was adjusted to the one from Lykartsis et al. that could be confirmed to fit [29].
3. Two additional questions were added to the post-experiment questionnaire: “How suitable do you consider a corporeal description of this music excerpt by movement according to musical contour?” and “How suitable do you consider a corporeal description of this music excerpt by movement according to rhythm?” (see S2 Fig). These two questions should provide additional information about the preference for certain movement patterns.

### Results and Discussion

#### Similarity between Movements and GEMS for Second Experiment

As you can observe from Tables 8 and 9, no significant features were found for *transcendence*. Unlike the first experiment, music featuring *wonder* was described by slow (median_dist_midcrosses) movements with irregular and longer backward phases (median_fall and std_fall). Though, *wonder* was related to regular backward phases at the same time (skewness_fall). Furthermore, gestures were of regular size (std_peak). As in Experiment 1 *power* is reflected by large movements but of varying size (std_peak) and speed (std_dist_midcrosses). *Tenderness* was related to slow motion (max_freq_hz and median_dist_midcrosses) of regular size and longer backward phases. *Nostalgia*, too, was performed by slow and regularly sized movements but with irregularly long forward phases. Similar to the first experiment, *peacefulness* was characterized by small movements. During the second experiment, additionally slow and regularly sized gestures were observed. *Joy*, however, stimulated regular, large and fast gestures. In the second experiment *sadness* was correlated to tempo being slow and irregular. Here, *tension* was characterized by fast but large movements. The gesture’s size was a significant feature during the first experiment, too.

**Table 8:** Fixed Effects Modeling Parameter Estimates for GEMS Ratings and their Principle Components of Second Experiment Predicted via Motion Features Related to *Size*, *Rate*, *Regularity* and *Smoothness* of the Movement.

Fixed Effects	estimate	SE	t-statistic	p-value
GEMS-9				
**transcendence**				
none	-	-	-	-
**wonder**				
intercept	56.5	5.9	9.5	.0000
std_fall_PC1	14.2	6.3	2.3	.0252
skewness_fall_PC1	-15.9	6.6	-2.4	.0172
std_peak_PC1	-32.6	6.6	-4.9	.0000
median_dist_midcrosses_PC2	17.2	6.7	2.6	.0111
median_fall_PC3	12.7	6.4	2.0	.0478
**power**				
intercept	23.1	4.2	5.5	<.0001
median_peak_PC1	28.7	7.4	3.9	.0002
std_dist_midcrosses_PC2	14.3	5.9	2.4	.0165
median_peak_PC2	21.5	7.7	2.8	.0061
std_peak_PC2	19.9	7.1	2.8	.0058
**tenderness**				
intercept	58.5	6.0	9.8	.0000
max_freq_hz	-16.8	5.8	-2.9	.0046
median_dist_midcrosses_PC1	16.7	7.8	2.1	.0336
skewness_fall_PC1	-18.4	6.9	-2.7	.0088
std_peak_PC1	-20.2	6.5	-3.1	.0022
median_fall_PC3	13.8	6.5	2.1	.0351
**nostalgia**				
intercept	39.3	5.2	7.5	<.0001
std_peak_PC1	-28.0	7.1	-4.0	.0001
median_dist_midcrosses_PC2	29.6	6.8	4.4	<.0001
std_rise_PC3	14.6	6.5	2.2	.0263
**peacefulness**				
intercept	52.6	6.2	8.4	<.0001
skewness_rise_PC1	17.1	7.5	2.3	.0232
median_peak_PC1	-20.7	8.9	-2.3	.0215
median_dist_midcrosses_PC2	24.0	7.1	3.4	.0010
std_peak_PC2	-27.3	8.7	-3.1	.0022
**joyful activation**				
intercept	40.4	5.3	7.6	<.0001
max_freq_hz	16.5	4.9	3.3	.0011
std_rise_PC1	-12.3	5.7	-2.1	.0341
std_fall_PC3	-18.8	5.8	-3.2	.0015
skewness_fall_PC3	18.0	6.1	3.0	.0037
median_peak_PC3	22.1	5.9	3.7	.0003
**sadness**				
intercept	21.1	5.6	3.8	.0002
median_dist_midcrosses_PC1	25.5	7.3	3.5	.0006
std_dist_midcrosses_PC1	11.7	6.4	1.8	.0705
std_fall_PC1	16.6	6.3	2.6	.0094
skewness_fall_PC1	-18.9	7.1	-2.7	.0086
std_fall_PC3	14.6	5.8	2.5	.0141
**tension**				
intercept	40.3	5.4	7.5	<.0001
volume	27.1	6.7	4.1	.0001
skewness_rise_PC1	-19.3	7.5	-2.6	.0116
median_dist_midcrosses_PC2	-31.0	6.9	-4.5	<.0001

**Table 9:** Fixed Effects Modeling Parameter Estimates for GEMS Ratings and their Principle Components of Second Experiment CONTINUED.

Fixed Effects	estimate	SE	t-statistic	p-value
Principle Components of GEMS-9				
**PC-1 relaxation**				
intercept	0.5	0.2	2.4	.0189
skewness_fall_PC1	-0.6	0.2	-2.5	.0153
std_peak_PC1	-1.0	0.2	-4.0	.0001
median_dist_midcrosses_PC2	0.8	0.2	3.2	.0018
skewness_dist_midcrosses_PC3	-0.5	0.2	-2.2	.0288
**PC-2 negative valence**				
intercept	0.2	0.2	1.1	.2887
median_dist_midcrosses_PC1	0.6	0.2	2.3	.0254
std_dist_midcrosses_PC1	0.5	0.2	2.2	.0274
std_rise_PC1	0.5	0.2	2.2	.0315
skewness_fall_PC3	-0.7	0.2	-3.2	.0016
median_peak_PC3	-1.1	0.2	-5.2	<.0001

**Fig 4:**
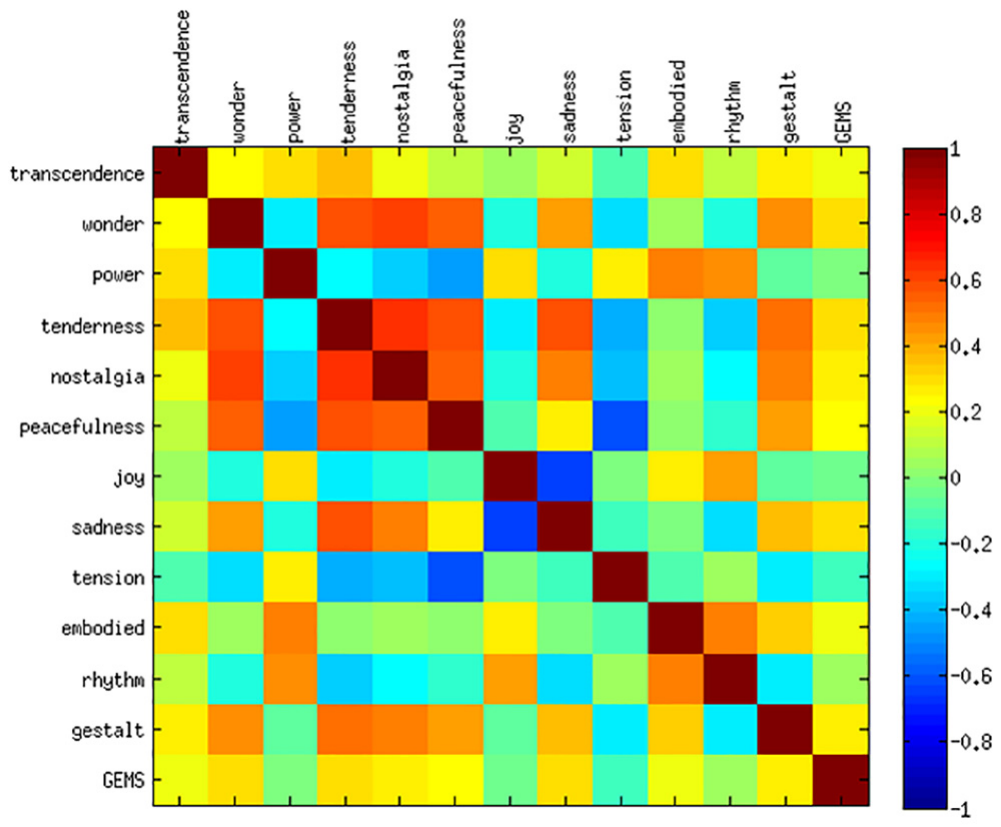
Spearman Correlation of GEMS and Description Preferences (Embodied (Gestalt or Rhythm), GEMS) for Second Experiment.

Confirming both of our hypotheses on description preferences, participants preferred GEMS and gestalt movements referring to melodic contour to describe their experience when they perceived *wonder, sadness, tenderness, peacefulness or nostalgia* (see Fig 4). For *joy* and *power* participants preferred embodied and rhythmic descriptions. For *tension* and *transcendence* were not related to description preference.

#### Prediction Results for Second Experiment

Table 10 shows that *power* and *tenderness* were also among the top prediction results for the second experiment. This time, however, *sadness*, *joy*, *peacefulness* and *wonder* scored much higher in terms of *R*^2^ whereas *nostalgia*, *tension* and *transcendence* were among the GEMS being most difficult to predict. Since for both, the training and test data set, a comparable amount of variance could be explained, there was no evidence for overfitting of the model to the data.

**Table 10:** R-Squared and RMSE for Second Experiment Ranked According to their *R*^2^ on the Training Set.

	rmse (training)	*R*^2^ (training)	p-value (training)	rmse (test)	*R*^2^ (test)
**GEMS-9**					
power	19.0	.494	<.0001	27.3	.375
tenderness	23.0	.385	<.0001	26.2	.388
sadness	23.1	.346	<.0001	29.2	.322
peacefulness	24.4	.345	<.0001	25.7	.303
wonder	23.2	.343	<.0001	25.0	.586
joyful activation	21.9	.325	<.0001	24.4	.276
nostalgia	25.1	.295	<.0001	26.3	.334
tension	24.8	.265	<.0001	27.3	.266
transcendence	-	-	-	-	-
**PCs of GEMS-9**					
PC-1 relaxation	.9	.330	<.0001	.9	.450
PC-2 negative valence	.9	.354	<.0001	.9	.368

GEMS ratings for the second experiment were similarly correlated like in the first one (see S1 Fig). Applying PCA to the GEMS rating data also resulted in two components explaining most of the variance in the data (according to the Elbow Method). Table 11 indicates that *relaxation* and *negative valence* were present as most dominant principle components. For *relaxation* loadings were again highly positive for *tenderness*, *peacefulness* and *nostalgia*, but highly negative loads for *tension*. The second PC got high negative loads for *joy*, but positive loads for *sadness*.

**Table 11:** Principle Component Loadings of GEMS ratings of Second Experiment.

GEMS feeling	PC-1 relaxation	PC-2 negative valence
tenderness	.781	.336
peacefulness	.778	.214
nostalgia	.745	.334
wonder	.717	.296
tension	-.697	.027
transcendence	.494	-.353
joy	.028	-.855
sadness	.343	.714
power	-.229	-.599

Notes: Components have been rotated using to the *varimax* method.

In contrast to the first experiment, estimates for the fixed effects (Table 9) show that *relaxation* was expressed by slow and regular movements while *negative valence* related mainly to small and slow movements. This time, prediction results (Table 10) indicate that for *relaxation* and *negative valence* about one third of the variance in data could be described by the fitted regression models. Since for both, the training and test data set, a comparable amount of variance could be explained, there was no evidence for overfitting of the model to the data.

#### Discussion

The second study confirmed that there were two principal components in the GEMS ratings: *relaxation* and *(positive/negative) valence*. This time, for *relaxation* and *valence* approximately same amounts of variance could be explained by the regression models. That might probably be due to the fact that for the second experiment *joyful* experiences could be predicted to a much better degree. Also *sadness* and *peacefulness* were predicted better, while for *transcendence* no regression model could be fitted since there were no significant features. In general, prediction results from the first experiment were slightly worse. That might be due to the chosen samples or the participants’ movement patterns or both. Another possibility is that participants did not keep up movement speed or size over the whole period of the musical excerpt and e.g. only moved half-time for fast music. That is why, they might sometimes not be able to describe the experience corporeally, though they rate it as more *powerful*. Hence, for ergonomic and biomechanical reasons, corporeal descriptions and GEMS ratings might drift apart (cf. [[12]pp. 112–114]). One countermeasure could be to inspect the motion data in different time windows and to chose the window featuring maximum speed or size, instead of simply averaging over the whole time. The extracted motion features already covered a good degree of rhythmic qualities but were ignorant of the course and direction of the movement expressed. These gestalt features could be particularly important when it comes to complex emotional expressions in music like *nostalgia*.

## General and Concluding Discussion

Results for both experiments showed that movements predicted both *arousal* and in Experiment 2 also *valence*. The quality of prediction for different degrees of GEMS also varied between experiments for *joy*, *sadness*, *transcendence*, *wonder* and *tension*, for others like *power* or *peacefulness* results could be reproduced. For many GEMS feelings, participants applied similar movement patterns across the two experiments (cf. Tables 4, 8 and 9).

These movement patterns often follow the similar principles reported in previous studies of [17] and [8]. There, *joy or happiness* was consistently associated with medium sound levels, high tempi and small timing variation. The size (large) of the movement (equaling sound level) and small timing variation were significant features for the second experiment. Furthermore, like both studies cited, we also showed that *sadness* was correlated with irregular and slow movements.

Considering the prediction results, there are some possible explanations for the better results of the more rhythm-related feelings (e.g. *power*, cf. Fig 4) over those requiring presumably more additional gestalt elements: Emotions like *nostalgia* and *transcendence* call for features that are less bound to rhythm but to the musical contour like directional and time series features. Acceleration data seems to not sufficiently cover *nostalgic* or *transcendent* gestures. As acceleration does not determine the absolute position in space, it is particularly difficult for slow but big gestures to be captured. This calls for applying different and more sophisticated motion sensor fusion techniques. Participants preferred the embodied description for the more *energetic* and *joyful* musical excerpts (cf. Figs 2 and 4) and hence, they might also not be trained to express certain feelings like *sadness* or *transcendence* in a corporeal way. Furthermore, there might be significant inter-individual differences in how participants are able to express feelings through movement that would also be an interesting topic of future investigations. Also, given the often reported high inter-individual variance of musical emotion, a much larger data set would be desirable. This would allow to model the different individual or stimulus specific sources of variance in the data.

### Conclusion

This study evaluated the predictive power of mobile-device generated acceleration data produced by free movement during music listening experience for the prediction of different degrees of the Geneva Emotion Music Scales (GEMS-9). The results show that participants considered the corporeal description of music as very suitable and produced movement data that could be used to predict emotion ratings. Hence, this study contributed to the envisioned use case of multimodel querying in two ways: First of all, it emphasizes the user need and acceptance for an innovative embodied access to MIR and MRS. Secondly, it showed that such an approach is technically feasible. Since GEMS that are more related to rhythm could be predicted better, observations suggest that there might still be a lot of hidden potential in additional movement features capturing direction and position in order to describe the gestalt of the movement.

## Supporting Information

S1 Fig Distribution of GEMS Ratings for Music Samples for 2nd Experiment. (ZIP)

S2 Fig App Screenshots. (ZIP)
